# Low Body Mass Index Increases Frailty Risk in Old–Old Adults: Findings From a 3‐Year Longitudinal Study

**DOI:** 10.1111/ggi.70474

**Published:** 2026-04-03

**Authors:** Kento Tabira, Daijo Shiratsuchi, Yuto Miyake, Hyuma Makizako

**Affiliations:** ^1^ Department of Physical Therapy, School of Health Sciences, Faculty of Medicine Kagoshima University Kagoshima Japan; ^2^ Graduate School of Health Sciences Kagoshima University Kagoshima Japan

**Keywords:** body mass index, frailty, high BMI, low BMI, old–old adults

## Abstract

**Objectives:**

Frailty risk increases with age. It is important to understand how body mass index (BMI) relates to changes in frailty risk among adults aged 75 years and older (old–old adults). We examined the longitudinal association between baseline BMI and changes in frailty risk over 3 years.

**Methods:**

This 3‐year retrospective cohort study included 515 old–old adults (median age 79.3 years; 45.0% male) who participated in the Latter‐Stage Older Persons Health Checkup in Amami City, Japan. Participants were categorized into three BMI groups: < 21.5, 21.5–24.9, and ≥ 25.0 kg/m^2^. Frailty risk was assessed using the Old–Old Questionnaire for Medical Checkups, a validated tool for assessing multidimensional frailty. Thirteen items were analyzed, excluding smoking and social support domains, to better examine the association between BMI and frailty risk. Higher scores indicate a greater frailty risk. We used linear mixed‐effects models to analyze the associations between BMI categories at baseline and changes in frailty risk.

**Results:**

The BMI < 21.5 kg/m^2^ group participants reported a greater annual increase in frailty risk compared with those in the BMI 21.5–24.9 kg/m^2^ group (*β* = 0.17, Standard error = 0.07, *p* = 0.014). The BMI ≥ 25.0 kg/m^2^ group participants reported no significant change in frailty risk compared with those in the BMI 21.5–24.9 kg/m^2^ group (*β* = −0.01, Standard error = 0.06, *p* = 0.821).

**Conclusion:**

Old–old adults with a BMI of ≤ 21.5 kg/m^2^ experienced a more pronounced increase in frailty risk over the 3‐year follow‐up period.

## Graphical abstract

We studied the longitudinal association between baseline BMI and changes in frailty risk over 3 years in old–old adults. Old–old adults with a BMI of ≤ 21.5 kg/m^2^ experienced a more pronounced increase in frailty risk than those with a BMI range of 21.5–24.9 kg/m^2^.

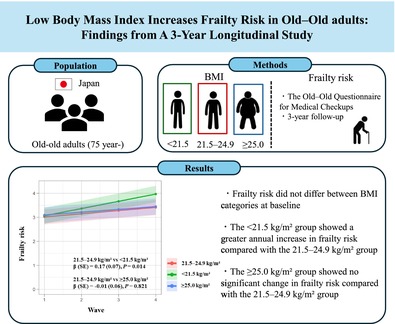

## 1 Introduction

Frailty is a geriatric condition characterized by heightened vulnerability and reduced capacity to preserve physiological homeostasis [1, 2]. It is a pathophysiological condition before the development of a serious disease associated with multiple adverse health outcomes such as falls, fractures, disability, entry to residential care, and mortality [3]. Among potential risk factors, body mass index (BMI) is a readily measurable indicator that reflects both nutritional and metabolic status [4]. Importantly, in the context of aging, BMI functions as an integrative indicator of nutritional status and energy reserves [5, 6], both of which are central to the frailty cycle [7]. In older adults, BMI reflects not only adiposity but also age‐related changes in body composition, including muscle loss and fat redistribution, thereby capturing the dual risks associated with both low and high body mass.

Several studies have reported an association between BMI and frailty risk. A systematic review of prospective cohort studies examining the longitudinal association between BMI and frailty risk established clear age‐specific risks [8]. Specifically, only a high BMI in middle age was associated with a significant increase in frailty risk in later life, whereas a low BMI was not significantly related to frailty risk. Conversely, among older adults, both high and low BMI were associated with a higher frailty risk. These findings underscore the importance of maintaining a BMI within a healthy range for one's age, suggesting that preventing excessive weight gain during middle age and avoiding both substantial weight gain and loss during old age are crucial for reduction of frailty risk. However, previous studies have commonly examined adults aged 65 years and older as a single group, and it is possible that in adults aged 75 years and older (old–old adults), the association between BMI and changes in frailty risk differs from that observed in these broader older populations.

Despite this foundational knowledge, evidence regarding the highest‐risk demographic, old‐old adults, remains critically limited. This population warrants special attention owing to the increased risk of adverse outcomes such as dementia [9], fractures [10], low quality of life [11], and mortality [12]. Age‐related changes in body composition and chronic energy‐depleting conditions become more pronounced in old–old adults, and BMI reflects not only adiposity but also nutritional status and energy reserve [13, 14]. Thus, the structure of the association between BMI and frailty risk may differ from that observed in younger older adults. Given that physiological changes associated with aging may increase frailty risk in this population [15, 16], longitudinal evidence tracking the impact of BMI on frailty risk changes in old‐old adults is needed. Moreover, old–old adults in Japan are recognized as a distinct population in public health practice, with healthcare programs and health checkups specifically targeting this age group, and the proportion of this population is rapidly increasing. Therefore, focusing on this population in epidemiological studies is particularly meaningful. Filling this gap is essential to developing targeted age‐specific strategies for reducing frailty risk.

Therefore, we studied the longitudinal association between baseline BMI and changes in frailty risk over 3 years in old–old adults. We hypothesized that, among old–old adults, lower BMI would be associated with an increased frailty risk over time, whereas higher BMI would not confer a comparable increase in frailty risk.

## 2 Methods

### 2.1 Study Design and Population

This was a retrospective cohort study of the residents of Amami City in Kagoshima Prefecture, Japan. Data were obtained from the Latter‐Stage Older Persons Health Check‐up conducted between April 2021 and December 2024. Latter‐Stage Older Persons Health Checkups are annual health screenings for detecting lifestyle diseases and frailty at an early stage in old‐old adults. All longitudinal data, including the baseline and follow‐up measurements, were extracted from these existing health check‐up records. Specifically, individuals who underwent the check‐up during Wave 1 (baseline: April 2021–March 2022) were enrolled, and their data from subsequent annual waves—Wave 2 (April 2022–March 2023), Wave 3 (April 2023–March 2024), and Wave 4 (April 2024–December 2024)—were tracked. Each participant attended the Latter‐Stage Older Persons Health Checkup on a single day within the respective waves. Participants with available baseline data (Wave 1) and at least one subsequent follow‐up (Waves 2–4) were included in the analysis. Although not all the participants had data available for every wave, all available repeated measurements were included in the longitudinal analysis.

**FIGURE 1:**
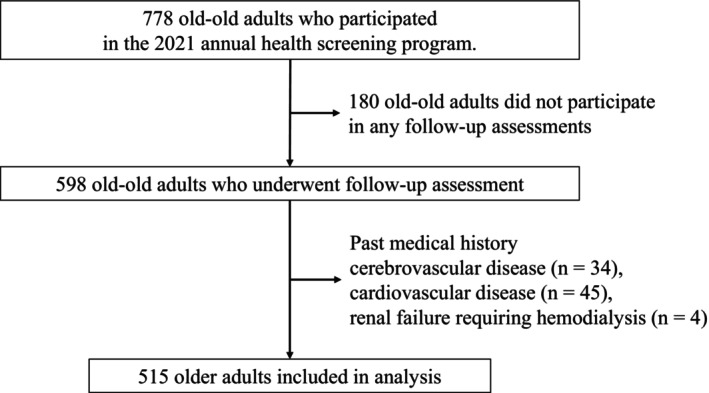
Flowchart of participants through the study.

Figure 1 shows the flow of participants throughout the study. At baseline, 778 participants were enrolled. Participants who did not participate in any follow‐up waves (Waves 2–4) were excluded from the analysis (*n* = 180). Participants with a history of cerebrovascular disease (*n* = 34), cardiovascular disease (*n* = 45), or end‐stage renal disease requiring hemodialysis (*n* = 4) were further excluded. The final analytical sample consisted of 515 participants. This study was approved by the Ethics Committee on Epidemiological and Related Studies, Sakuragaoka Campus, Kagoshima University (approval number: 240137), and was conducted in accordance with the principles of the Declaration of Helsinki.

### 2.2 Measurement and Classification of BMI

BMI was calculated by dividing the body weight (kg) by the square of height (m^2^). Participants were categorized into three BMI groups (< 21.5, 21.5–24.9, and ≥ 25.0 kg/m^2^) according to the target range for Japanese old–old adults recommended by the Dietary Reference Intakes for Japanese (2025) published by the Ministry of Health, Labor and Welfare [17]. This range was determined based on epidemiological evidence indicating the lowest all‐cause mortality and other relevant health factors [18, 19, 20].

### 2.3 Assessment of Frailty Risk

Frailty risk was assessed using the Questionnaire for Medical Checkup of Old–Old (QMCOO), which was developed to evaluate multidimensional frailty [21]. The questionnaire consists of 15 items across 10 domains: general health, mental health, dietary habits, oral function, weight loss, physical function and falls, cognitive function, cigarette smoking, social participation and social support. The weight‐loss item assesses body weight changes during the last 6 months. As this item captures short‐term weight change, whereas baseline BMI reflects body size at the time of assessment, the two measures differ in both their temporal reference and the underlying constructs they are intended to measure. Therefore, they were treated as conceptually distinct in the analysis. Scoring of the QMCOO was conducted according to a previous study [22], and the scores assigned to each item and response are presented in Table S1. Previous research demonstrated that the QMCOO shows strong agreement with established frailty assessment tools commonly used in Japan [22, 23]. The primary outcome of this study was longitudinal change in the QMCOO‐based frailty risk. Frailty onset during follow‐up was not treated as an endpoint; instead, changes in frailty risk were evaluated continuously over time.

To capture changes in frailty risk across BMI categories in greater detail, we excluded the smoking and social support domains from the primary outcome. These domains were excluded for conceptual reasons rather than statistical considerations, as they are less likely to vary longitudinally according to BMI categories in old–old adults. The resulting 13‐item QMCOO score was calculated by summing the scores for each item (range, 0–13), with higher scores indicating higher frailty risk. In addition, subscale scores were calculated for physical and cognitive functions. These subscales constitute components of frailty risk and were additionally analyzed as secondary outcomes to examine domain‐specific associations. The physical score was defined as the sum of three items related to physical function and falls: perceived decline in walking speed, history of falls last year, and participation in exercise at least once a week. Each item was scored on a 0–3 point scale. The cognitive score was assessed using two items: the presence of informant‐reported memory problems and temporal disorientation, with the total score ranging from 0 to 2 points.

### 2.4 Covariates

Covariates included age, sex, hypertension, diabetes, dyslipidemia, cigarette smoking, and social support at baseline. Covariates were selected based on epidemiological evidence indicating their relevance to both BMI and frailty risk in older adults [24, 25, 26, 27, 28]. Hypertension was defined as systolic blood pressure (SBP) ≥ 140 mmHg, diastolic blood pressure (DBP) ≥ 90 mmHg, or use of blood pressure‐lowering medications. Individuals with diabetes mellitus were classified based on a hemoglobin A1c (HbA1c) level of ≥ 6.5% or the use of glucose‐lowering medications. Dyslipidemia was defined as a low‐density lipoprotein cholesterol (LDL‐C) level of ≥ 140 mg/dL, a high‐density lipoprotein cholesterol (HDL‐C) level of < 40 mg/dL, a triglyceride level of ≥ 150 mg/dL, or the use of lipid‐lowering medications [29]. Information on cigarette smoking status and social support was assessed using the QMCOO [21]. Smoking status [24, 26] and social support [27, 28] were instead included as covariates in the analytical models to account for their independent effects on frailty risk.

### 2.5 Statistical Analysis

Continuous variables for descriptive statistics are presented as mean (standard deviation [SD]) or median (interquartile range [IQR]). Categorical variables are presented as numbers (percentages). Comparisons of the baseline characteristics were performed with one‐way analysis of variance or Kruskal–Wallis rank sum tests for continuous variables and *χ* ^2^ tests for categorical variables.

We next analyzed the associations of BMI categories at baseline with frailty risk, physical score, and cognitive score. We used linear mixed‐effect models to calculate the regression coefficients (β), standard errors (SE), and *p* values with the 21.5–24.9 kg/m^2^ group as the reference. Linear mixed‐effects models were selected because they allow flexible modeling of repeated measurements, accommodate unbalanced data due to missing observations, and provide straightforward interpretation of mean longitudinal changes [30]. In each model, repeated measurements of frailty risk across all waves were included as the primary dependent variable. In secondary analyses, repeated measurements of the physical score and cognitive score were included as secondary dependent variables. The fixed effects included BMI category, wave, the interaction between BMI category and wave, and covariates. Wave was treated as a continuous variable in the models. Random effects for both the intercept and slope were included to account for interindividual differences at baseline and in the rate of change over time. The coefficient of the wave represents the overall annual change in the outcome, whereas the coefficients of the interaction terms indicate differences in the annual change relative to the reference group. In addition, the estimated marginal means of each outcome for each BMI category in each wave were calculated using 95% confidence intervals derived from standard errors.

Several sensitivity analyses were performed to evaluate the robustness of the findings with respect to the definition of frailty risk and the BMI classification applied: [1] using the total score of the 15‐item QMCOO as the measure of frailty risk (without adjustment for cigarette smoking status and social support), [2] using frailty risk based on 12 items, following previous studies [31], excluding general health (composed of two items) and cigarette smoking (without adjustment for social support), [3] using BMI categories defined according to the Global Leadership Initiative on Malnutrition (GLIM) criteria [32] (< 20.0, 20.0–24.9, and ≥ 25.0 kg/m^2^), [4] using BMI categories with obesity defined as BMI ≥ 30.0 [33] (< 21.5, 21.5–29.9, and ≥ 30.0 kg/m^2^) and [5] using participants with complete data across all four waves (*n* = 270).

In all models, missing data were assumed to be randomly missed. The sensitivity analysis, restricted to participants with complete data, was specifically conducted to assess the potential impact of omission due to death, incident disease, or functional decline. All statistical analyses were performed using the R software (version 4.5.1). All *p* values were two‐sided, and statistical significance was defined as *p* < 0.05.

## 3 Results

### 3.1 Baseline Characteristics of Study Participants

The baseline characteristics (wave 1) of study participants according to the BMI category are presented in Table 1. The median age of all participants was 79.3 (77.2–82.1) years, and 232 participants (45.0%) were male. The mean BMI (SD) was 23.7 (3.0) kg/m^2^, with 260 participants (50.5%) in the 21.5–24.9 kg/m^2^ group, 110 (21.4%) in the < 21.5 kg/m^2^ group, and 145 (28.2%) in the ≥ 25.0 kg/m^2^ group.

**TABLE 1:** Characteristics of participants (*n* = 515).

	All (*n* = 515)	21.5–24.9 kg/m^2^ (*n* = 260)	< 21.5 kg/m^2^ (*n* = 110)	≥ 25.0 kg/m^2^ (*n* = 145)	*p* ^a^
Age, median (IQR), years	79.3 (77.2–82.1)	79.0 (76.9–82.1)	79.1 (77.1–81.2)	79.8 (77.6–82.7)	0.099
Male, *n* (%)	232 (45.0%)	120 (46.2%)	45 (40.9%)	67 (46.2%)	0.616
BMI, mean (SD), kg/m^2^	23.7 (3.0)	23.3 (1.0)	20.0 (1.3)	27.4 (2.3)	< 0.001
SBP, mean (SD), mmHg	132.1 (15.1)	131.1 (15.3)	130.1 (15.8)	135.5 (13.7)	0.005
DBP, mean (SD), mmHg	71.7 (9.8)	71.4 (9.6)	70.1 (9.0)	73.5 (10.4)	0.016
Hypertension, *n* (%)	336 (65.2%)	159 (61.2%)	61 (55.5%)	116 (80.0%)	< 0.001
Diabetes, *n* (%)	59 (11.5%)	30 (11.5%)	7 (6.4%)	22 (15.2%)	0.091
Dyslipidaemia, *n* (%)	313 (60.8%)	169 (65.0%)	48 (43.6%)	96 (66.2%)	< 0.001
Cigarette Smoking, *n* (%)	17 (3.3%)	9 (3.5%)	7 (6.4%)	1 (0.7%)	0.042
Social support, *n* (%)	11 (2.1%)	6 (2.3%)	2 (1.8%)	3 (2.1%)	0.955
Laboratory data					
HbA1c, median (IQR), %	5.6 (5.4–5.8)	5.6 (5.4–5.9)	5.4 (5.3–5.7)	5.7 (5.4–6.0)	< 0.001
LDL‐C, median (IQR), mmol/L	116.0 (97.0–132.0)	118.0 (97.0–133.0)	115.0 (98.5–133.3)	114.0 (97.0–130.0)	0.522
HDL‐C, median (IQR), mmol/L	57.0 (48.0–67.0)	56.0 (47.0–66.0)	64.0 (54.8–74.0)	54.0 (46.0–64.0)	< 0.001
Triglycerides, median (IQR), mmol/L	97.0 (73.0–134.0)	102.0 (77.5–138.0)	81.5 (62.8–102.3)	103.0 (80.5–153.5)	< 0.001
Frailty score, median (IQR)	2 (1–3)	2 (1–3)	2 (1–3)	2 (1–3)	0.568
Physical score, median (IQR)	1 (0–1)	1 (0–2)	1 (0–1)	1 (0–2)	0.077
Cognitive score, median (IQR)	0 (0–0)	0 (0–0)	0 (0–0)	0 (0–0)	0.604

Abbreviations: BMI, body mass index; DBP, diastolic blood pressure; HDL‐C, high‐density lipoprotein cholesterol; IQR, interquartile range; LDL‐C, low‐density lipoprotein cholesterol; SBP, systolic blood pressure; SD, standard deviation.

^a^ Comparisons of baseline characteristics among the three groups are performed using one‐way ANOVA or Kruskal–Wallis rank sum tests for continuous variables, and *χ* ^2^ tests for categorical variables.

Participants in the ≥ 25.0 kg/m^2^ group had the mean SBP, DBP, HbA1c, and triglycerides, as well as the highest prevalence of hypertension and dyslipidaemia, whereas HDL‐C was the lowest in this group. In contrast, the proportion of cigarette smokers was highest in the < 21.5 kg/m^2^ group. No significant differences were noted in frailty risk, physical score, or cognitive score between the groups at baseline.

### 3.2 Associations of BMI Category With Frailty Risk

**FIGURE 2:**
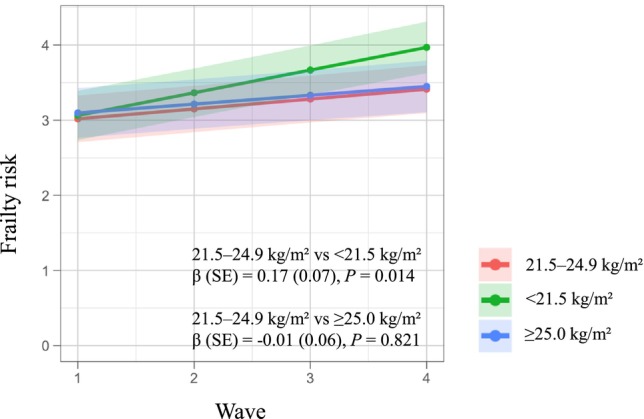
Predicted trajectories of frailty risk based on the BMI categories (< 21.5, 21.5–24.9, and ≥ 25.0 kg/m^2^). The intercept of each line indicates the baseline value, and the slope represents the annual change. The shaded areas around the lines represent 95% confidence intervals. β, SE, and *p* values indicate the difference in slopes of < 21.5 and ≥ 25.0 kg/m^2^ groups compared with the 21.5–24.9 kg/m^2^ group. BMI, body mass index; SE, standard error.

Across the follow‐up period, frailty risk increased on average by 0.13 points per year (SE = 0.04 and *p* < 0.001). Figure 2 shows the predicted trajectories of frailty risk by BMI category, along with estimated associations relative to the 21.5–24.9 kg/m^2^ group. The < 21.5 kg/m^2^ group showed a greater annual increase in frailty risk compared with the 21.5–24.9 kg/m^2^ group (*β* = 0.17, SE = 0.07, and *p* = 0.014). In contrast, the ≥ 25.0 kg/m^2^ group showed no significant change in frailty risk compared with the 21.5–24.9 kg/m^2^ group (*β* = −0.01, SE = 0.06, and *p* = 0.821).

**FIGURE 3:**
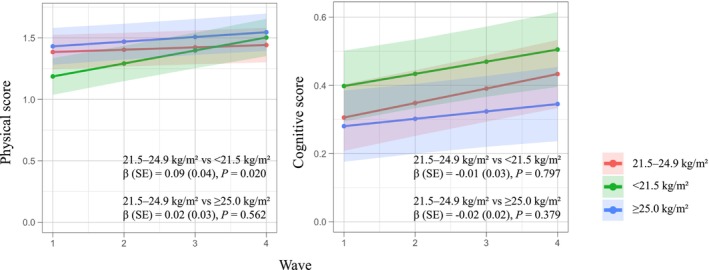
Predicted trajectories of physical and cognitive scores based on the BMI categories (< 21.5, 21.5–24.9, and ≥ 25.0 kg/m^2^). The intercept of each line indicates the baseline value, and the slope represents the annual change. The shaded areas around the lines represent 95% confidence intervals. β, SE, and *p* values indicate the difference in slopes of < 21.5 kg/m^2^ and ≥ 25.0 kg/m^2^ groups compared with the 21.5–24.9 kg/m^2^ group. BMI, body mass index; SE, standard error.

Over the follow‐up period, physical scores did not show a significant change (*β* = 0.02, SE = 0.02, and *p* = 0.333), whereas cognitive scores increased on average by 0.04 points per year (SE = 0.01, *p* = 0.003). Figure 3 shows the predicted trajectories of physical and cognitive scores by BMI category, along with the estimated associations relative to the 21.5–24.9 kg/m^2^ group. The < 21.5 kg/m^2^ group showed a greater annual increase in physical score compared with the 21.5–24.9 kg/m^2^ group (*β* = 0.09, SE = 0.04, and *p* = 0.020). In contrast, the ≥ 25.0 kg/m^2^ group did not show a significantly different increase in physical score compared with the 21.5–24.9 kg/m^2^ group (*β* = 0.02, SE = 0.03, and *p* = 0.562). The < 21.5 kg/m^2^ group showed no significant difference in cognitive score change compared with the 21.5–24.9 kg/m^2^ group (*β* = −0.01, SE = 0.03, *p* = 0.797). Similarly, the ≥ 25.0 kg/m^2^ group showed no significant difference (*β* = −0.02, SE = 0.02, *p* = 0.379).

### 3.3 Sensitivity Analysis

Sensitivity analyses were generally consistent with the main findings, showing similar results for alternative QMCOO‐based frailty risk, different BMI definitions, and analyses restricted to participants who completed all four survey waves (Figures S1–S5).

## 4 Discussion

This study examined the 3‐year longitudinal associations between baseline BMI and changes in frailty risk in old–old adults. Overall, the frailty risk increased over the follow‐up period. Although frailty risk did not differ between BMI categories at baseline, the < 21.5 kg/m^2^ group demonstrated a more pronounced increase in frailty risk during the follow‐up period compared with the 21.5–24.9 kg/m^2^ group. Analyses of subscales revealed that the < 21.5 kg/m^2^ group showed a pronounced increase in the physical score compared with the 21.5–24.9 kg/m^2^ group, whereas no significant differences in cognitive function scores were observed among the BMI categories.

Previous longitudinal studies have reported a U‐shaped or J‐shaped association between BMI and frailty risk [4, 34, 35, 36]. However, unlike previous studies, frailty risk increased only in those with a BMI < 21.5 kg/m^2^. This discrepancy may reflect age‐related differences, as the current study specifically targeted old–old adults, as well as differences in BMI categorization.

Previous studies have suggested that the association between BMI and frailty risk varies with age [8, 37]. Participants in the current study were aged 75 years or older, whereas previous studies targeted adults aged 60 or 65 years and older. A low BMI has been interpreted in previous studies as a marker of reduced muscle mass and undernutrition, which, when combined with diminished physiological reserves, typically observed in old–old adults, may plausibly contribute to increased frailty risk and physical scores [15, 38, 39, 40]. Although muscle mass and nutritional status were not directly assessed in the present study, this interpretation is consistent with the existing epidemiological and clinical evidence. In contrast, among older adults with BMI ≥ 25.0 kg/m^2^, maintaining a certain amount of body fat may be associated with greater energy reserves and preservation of physiological reserve capacity in old–old adults [41], which could partly explain the observed attenuation in frailty risk [42].

In this study, BMI categories were defined based on the Dietary Reference Intake for the Japanese (2025) [17]. This categorization differs from conventional international standards (< 18.5, 18.5–29.9, ≥ 30 kg/m^2^) [33] and the GLIM criteria (< 20.0 kg/m^2^) [32], resulting in a shift in the classification of individuals with lower and higher BMI. Notably, the < 21.5 kg/m^2^ group included individuals who would have been considered normal weight (18.5–21.4 kg/m^2^) under conventional standards. Despite this, old–old adults with BMI < 21.5 kg/m^2^ showed a more pronounced increase in frailty risk over the follow‐up period, indicating that not only those who were underweight (BMI < 18.5 kg/m^2^) but also individuals with a BMI of 18.5–21.4 had a greater increase in frailty risk compared with those in the 21.5–24.9 kg/m^2^ range. Importantly, consistent associations were observed across multiple sensitivity analyses using alternative BMI cutoffs, supporting the conclusion that the observed longitudinal increase in frailty risk among old–old adults with relatively low BMI was not driven by the specific BMI classification. Among Japanese older adults, individuals with a BMI ≥ 30 kg/m^2^ are relatively rare [43], and the ≥ 25.0 kg/m^2^ BMI group in the current study primarily comprised those who were overweight (25.0–30.0 kg/m^2^). Consequently, an increased frailty risk may have been less likely to be observed in the ≥ 25.0 kg/m^2^ group compared with the 21.5–24.9 kg/m^2^ group.

The annual increase in the frailty risk score observed in this study was 0.13 points/year overall. In the BMI of < 21.5 kg/m^2^ group, adding the difference from the reference group (0.17 points/year) resulted in an annual increase of approximately 0.3 points. Considering that previous studies have defined prefrailty as a score of 3 and frailty as a score exceeding 4 out of 15 items [44], this represents a nonnegligible cumulative change. Given that frailty is associated with a range of adverse health outcomes, the findings of this study highlight the significance of identifying and closely monitoring old–old adults with BMI < 21.5 kg/m^2^. Those with a BMI < 21.5 kg/m^2^ are at higher frailty risk, and even individuals within the “normal” BMI range of 18.5–21.5 kg/m^2^ may represent a potential high‐risk group. Therefore, careful assessment and follow‐up may be warranted not only for old–old adults with a BMI < 18.5 kg/m^2^, but also for those with a low BMI ranging from 18.5 to 21.5 kg/m^2^. Further longitudinal studies are warranted to better contextualize the magnitude of BMI‐frailty associations.

The current study had several limitations. First, frailty risk was primarily assessed using subjective measures, which may be influenced by the participants' cognitive and psychological states and may have limited reproducibility and concordance with objective indicators. Second, the study population was limited to older Japanese adults, whose BMI distribution and body composition characteristics may differ from those of other populations [43], warranting caution in generalizing the findings. Third, certain data were excluded because of participant dropouts during follow‐up, which may have introduced a selection bias depending on the characteristics of those lost to follow‐up. Fourth, reverse causation cannot be ruled out in either the < 21.5 kg/m^2^ or ≥ 25.0 kg/m^2^ groups. Specifically, unintentional weight loss or weight changes due to underlying frailty or preclinical disease may have preceded the observed BMI status at baseline, which should be considered when interpreting the longitudinal associations. In addition, this study examined longitudinal associations between baseline BMI and frailty risk rather than the onset of frailty; therefore, incident frailty could not be determined. Finally, the covariates included in the analyses were limited, and other potential factors affecting frailty risk, such as educational level [45] and income [46], could not be fully accounted for. Thus, residual confounding by unmeasured factors cannot be excluded, which may have influenced the observed results in both the < 21.5 and ≥ 25.0 kg/m^2^ groups.

## 5 Conclusion

This study examined the longitudinal association between BMI at baseline and changes in frailty risk and demonstrated that old–old adults with a BMI of ≤ 21.5 kg/m^2^ experienced a more pronounced increase in frailty risk over the 3‐year follow‐up period than those with a BMI range of 21.5–24.9 kg/m^2^.

## Author Contributions

**Kento Tabira:** conceptualization, methodology, data curation, formal analysis, visualization, writing – original draft. **Daijo Shiratsuchi:** conceptualization, data curation, writing – review and editing. **Yuto Miyake:** conceptualization, data curation, writing – review and editing. **Hyuma Makizako:** conceptualization, data curation, project administration, resources, supervision, writing – review and editing.

## Funding

This study was conducted with financial support from the NEC Corporation and was supported by the MHLW Research on Policy Planning and Evaluation Program (24FA1005).

## Disclosure

The authors have nothing to report.

## Supporting information


**Table S1:** English translation of the Questionnaire for Medical Checkup of Old‐Old and assigned scores.
**Figure S1:** Predicted trajectories of frailty risk based on BMI categories from the 15‐item total score.
**Figure S2:** Predicted trajectories of frailty risk based on BMI categories from 12 items identified previous studies.
**Figure S3:** Predicted trajectories of frailty risk based on BMI categories defined according to GLIM criteria (< 20.0, *n* = 42; 20.0–24.9, *n* = 339; ≥ 25.0, *n* = 134).
**Figure S4:** Predicted trajectories of frailty risk based on BMI categories defined according to WHO criteria for obesity (< 21.5, *n* = 110; 21.5–29.9, *n* = 383; ≥ 30.0, *n* = 22).
**Figure S5:** Predicted trajectories of frailty risk based on BMI categories using complete case analysis (< 21.5, *n* = 53; 21.5–24.9, *n* = 144; ≥ 25.0, *n* = 73).

## Data Availability

Research data are not shared.
