# Supplementary material for: Low Body Mass Index Increases Frailty Risk in Old–Old Adults: Findings From a 3‐Year Longitudinal Study
Source: Geriatr Gerontol Int. 2026 Apr 3;26(4):e70474. doi: 10.1111/ggi.70474 (PMC13048879; doi:10.1111/ggi.70474)
Supplement: Supplementary file 1 — Table S1: English translation of the Questionnaire for Medical Checkup of Old‐Old and assigned scores. Figure S1: Predicted trajectories of frailty risk based on BMI categories from the 15‐item total score. Figure S2: Predicted trajectories of frailty risk based on BMI categories from 12 items identified previous studies. Figure S3: Predicted trajectories of frailty risk based on BMI categories defined according to GLIM criteria (< 20.0, n = 42; 20.0–24.9, n = 339; ≥ 25.0, n = 134). Figure S4: Predicted trajectories of frailty risk based on BMI categories defined according to WHO criteria for obesity (< 21.5, n = 110; 21.5–29.9, n = 383; ≥ 30.0, n = 22). Figure S5: Predicted trajectories of frailty risk based on BMI categories using complete case analysis (< 21.5, n = 53; 21.5–24.9, n = 144; ≥ 25.0, n = 73). [file GGI-26-0-s001.docx]

**Supplemental materials**

Table and Figure of contents

Table S1: English translation of the Questionnaire for Medical Checkup of Old-Old and assigned scores

Figure S1: Predicted trajectories of frailty risk based on BMI categories from the 15-item total score

Figure S2: Predicted trajectories of frailty risk based on BMI categories from 12 items identified previous studies

Figure S3: Predicted trajectories of frailty risk based on BMI categories defined according to GLIM criteria (<20.0, n = 42; 20.0–24.9, n = 339; ≥25.0, n = 134)

Figure S4: Figure S4: Predicted trajectories of frailty risk based on BMI categories defined according to WHO criteria for obesity (<21.5, n = 110; 21.5–29.9, n = 383; ≥30.0, n = 22)

Figure S5: Predicted trajectories of frailty risk based on BMI categories using complete case analysis (<21.5, n = 53; 21.5–24.9, n = 144; ≥25.0, n = 73)

**Table S1. English translation of the Questionnaire for Medical Checkup of Old-Old and assigned scores**

| No. | Domain | Question | Score |
| --- | --- | --- | --- |
| Q1 | General health | How is your health condition? | Excellent/good/fair = 0;  poor/very poor = 1 |
| Q2 | Mental health | Are you satisfied with your daily life? | Satisfied/moderately satisfied = 0; moderately dissatisfied/dissatisfied = 1 |
| Q3 | Dietary habits | Do you eat three times a day? | Yes = 0; no = 1 |
| Q4 | Oral function | Do you have any difficulties eating tough foods compared to 6 months ago? | Yes = 1; no = 0 |
| Q5 |  | Have you choked on your tea or soup recently? | Yes = 1; no = 0 |
| Q6 | Weight loss | Have you lost 2 kg or more in the past 6 months? | Yes = 1; no = 0 |
| Q7 | Physical function and falls | Do you think you walk slower than before? | Yes = 1; no = 0 |
| Q8 |  | Have you experienced a fall in the past year? | Yes = 1; no = 0 |
| Q9 |  | Do you go for a walk for your health at least once a week? | Yes = 0; no = 1 |
| Q10 | Cognitive function | Do your family or your friends point out your memory loss? For example, “You ask the same question over and over again.” | Yes = 1; no = 0 |
| Q11 |  | Do you find yourself not knowing today's date? | Yes = 1; no = 0 |
| Q12 | Cigarette smoking | Do you smoke? | Yes = 1; no/I quit = 0 |
| Q13 | Social participation | Do you go out at least once a week? | Yes = 0; no = 1 |
| Q14 |  | Do you keep regular communication with your family and friends? | Yes = 0; no = 1 |
| Q15 | Social support | When you are not feeling well, do you have anyone you can talk with? | Yes = 0; no = 1 |


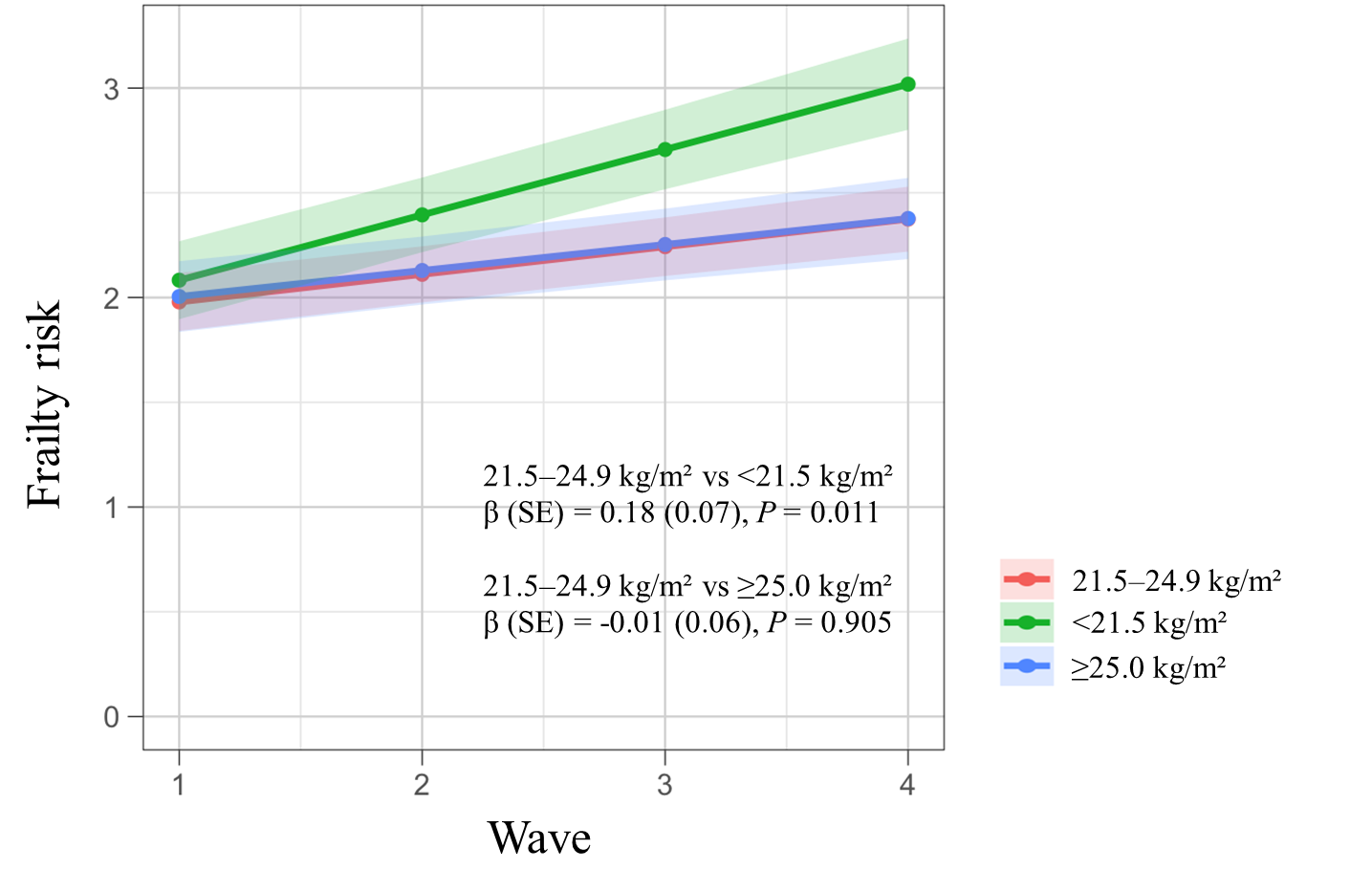


**Figure S1: Predicted trajectories of frailty risk based on BMI categories from the 15-item total score**

The intercept of each line indicates the baseline value, and the slope represents the annual change. The shaded areas around the lines represent the 95% confidence intervals. β, SE, and *P*-values indicate the difference in slopes of <21.5 kg/m² and ≥25.0 kg/m² groups compared with the 21.5-24.9 kg/m² group. BMI, Body mass index; SE: standard errors.


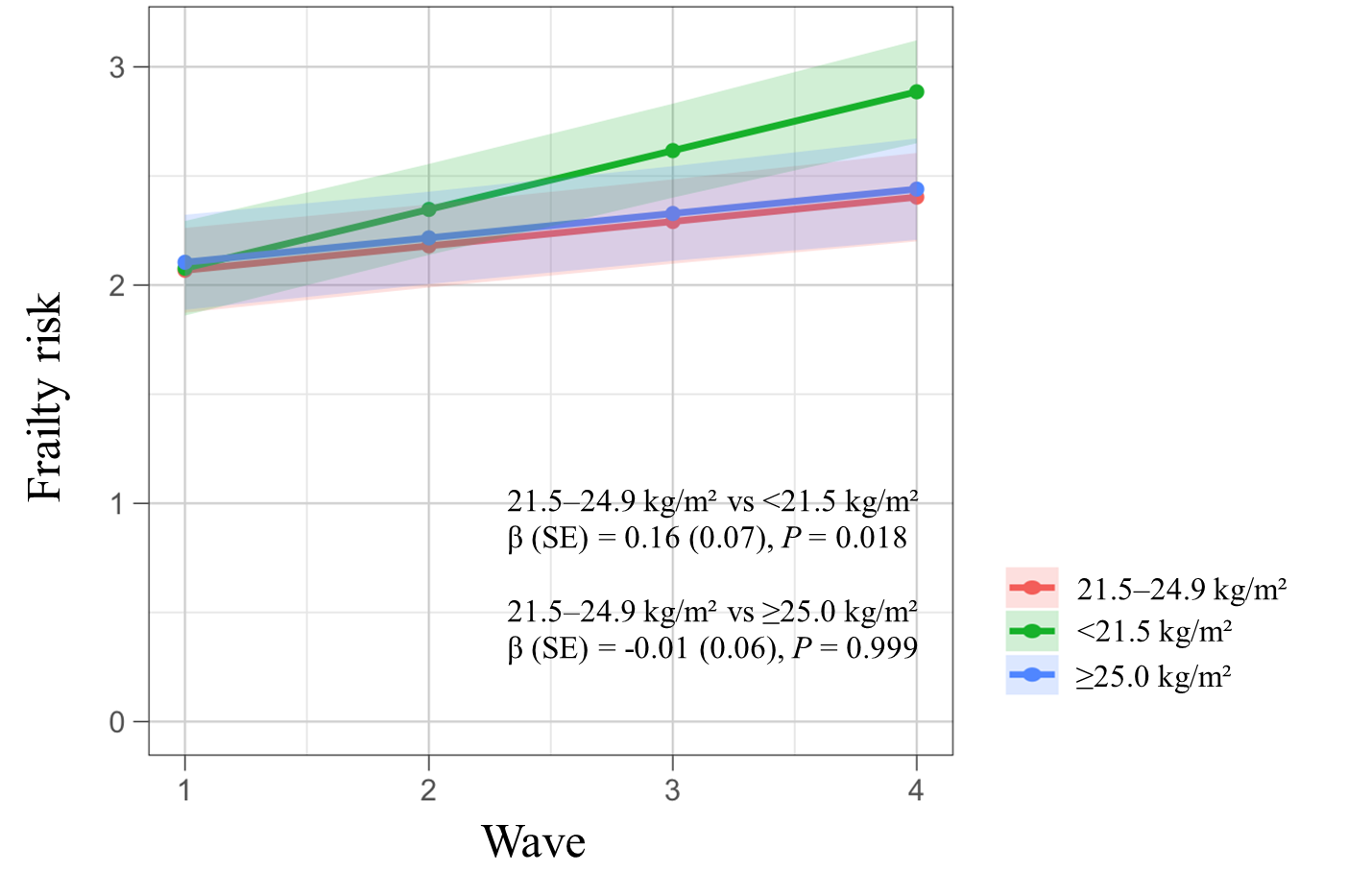


**Figure S2: Predicted trajectories of frailty risk based on BMI categories from 12 items identified previous studies**

The intercept of each line indicates the baseline value, and the slope represents the annual change. The shaded areas around the lines represent the 95% confidence intervals. β, SE, and *P*-values indicate the difference in slopes of <21.5 kg/m² and ≥25.0 kg/m² groups compared with the 21.5-24.9 kg/m² group. BMI, Body mass index; SE: standard errors.


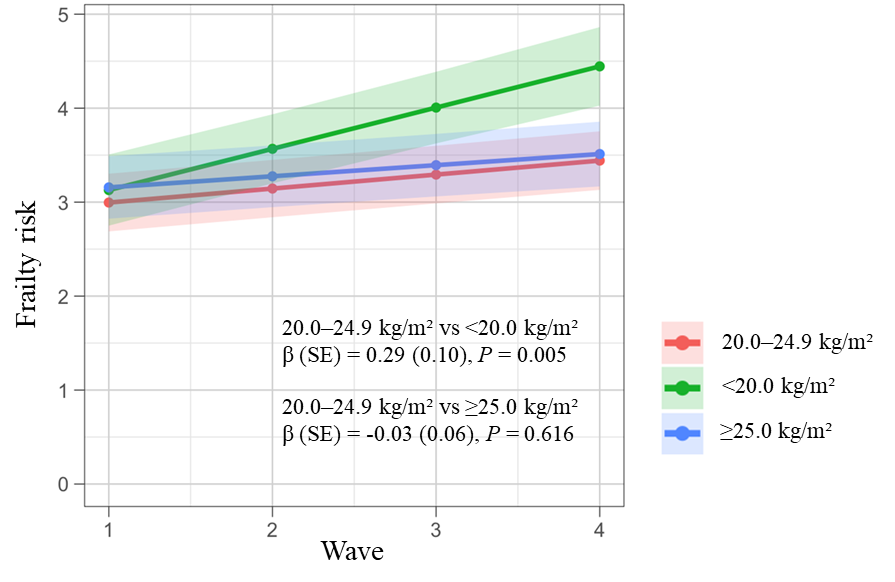


**Figure S3: Predicted trajectories of frailty risk based on BMI categories defined according to GLIM criteria (<20.0, n = 42; 20.0–24.9, n = 339; ≥25.0, n = 134)**

The intercept of each line indicates the baseline value, and the slope represents the annual change. The shaded areas around the lines represent the 95% confidence intervals. β, SE, and *P*-values indicate the difference in slopes of <20.0 kg/m² and ≥25.0 kg/m² groups compared with the 20.0-24.9 kg/m² group. BMI, Body mass index; SE: standard errors.


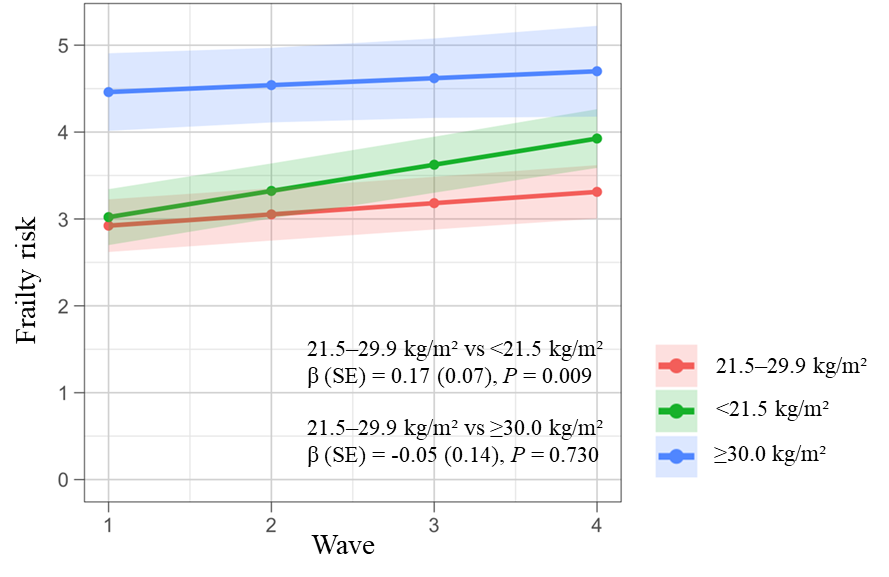


**Figure S4: Predicted trajectories of frailty risk based on BMI categories defined according to WHO criteria for obesity (<21.5, n = 110; 21.5–29.9, n = 383; ≥30.0, n = 22)**

The intercept of each line indicates the baseline value, and the slope represents the annual change. The shaded areas around the lines represent the 95% confidence intervals. β, SE, and *P*-values indicate the difference in slopes of <21.5 kg/m² and ≥30.0 kg/m² groups compared with the 21.5-29.9 kg/m² group. BMI, Body mass index; SE: standard errors.


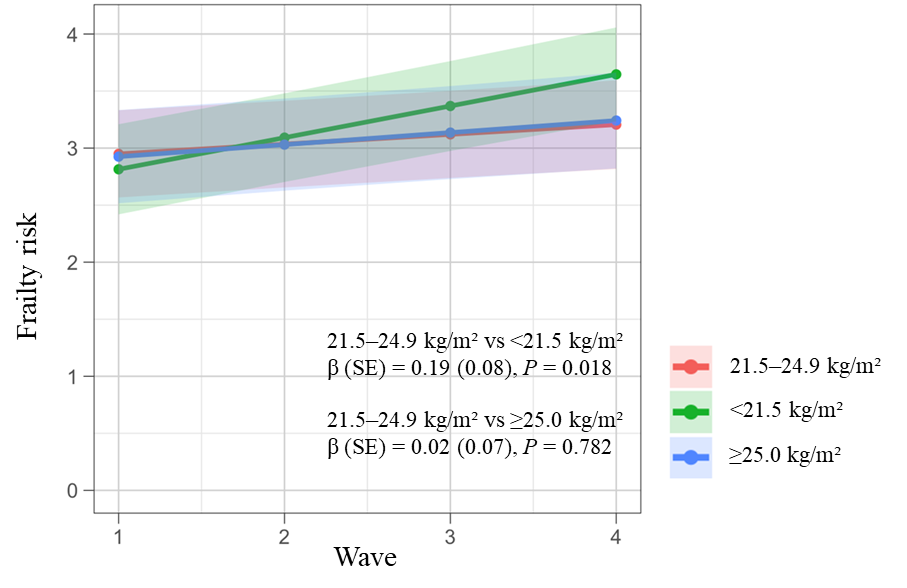


**Figure S5: Predicted trajectories of frailty risk based on BMI categories using complete case analysis (<21.5, n = 53; 21.5–24.9, n = 144; ≥25.0, n = 73)**

The intercept of each line indicates the baseline value, and the slope represents the annual change. The shaded areas around the lines represent the 95% confidence intervals. β, SE, and *P*-values indicate the difference in slopes of <21.5 kg/m² and ≥25.0 kg/m² groups compared with the 21.5-24.9 kg/m² group. BMI, Body mass index; SE: standard errors.
